# Commentary: Mesenchymal stem cells in the treatment of spinal cord injury: mechanisms, current advances and future challenges

**DOI:** 10.3389/fimmu.2024.1354118

**Published:** 2024-06-11

**Authors:** Sabino Luzzi, Antonio Crovace, Alberto M. Crovace

**Affiliations:** ^1^ Department of Clinical-Surgical, Diagnostic and Pediatric Sciences, University of Pavia, Pavia, Italy; ^2^ Department of Emergency and Organ Transplantation, School of Medicine, University of Bari Aldo Moro, Bari, Italy; ^3^ Department of Veterinary Medicine, University of Sassari, Sassari, Italy

**Keywords:** spinal cord injury, mesenchymal stem cells, axon regeneration, microglia, macrophages

## Background

We are writing a commentary after reading with great interest the article entitled *“Mesenchymal stem cells in the treatment of spinal cord injury: Mechanisms, current advances and future challenges”* by Dr. Xia and co-workers, published in the Frontiers in Immunology in February 2023 (1).

The author presented a comprehensive narrative review of the molecular mechanisms and therapeutic interventions for spinal cord injury (SCI), highlighting current and future potential applications of stem cell therapies, including mesenchymal stem cells (MSCs).

This is a complex and fascinating topic and I congratulate the author for the level of precision with which he has presented all the pros, cons, limitations, and challenges associated with the implementation of mesenchymal stem cell therapies for SCI.

MSCs have some well-known advantages over other types of stem cells, such as their multipotency, immunomodulatory capabilities and ease of isolation and culture.

With the aim of stimulating discussion and enhancing the value of the study, some additional considerations regarding the feasibility, efficacy, and safety of xenogeneic ovine bone marrow mesenchymal stem cells transplantation in SCI are reported here.

## Xenogeneic mesenchymal stem cells for SCI

We would like to draw attention to further innovative aspects of the potential applications of MSCs. Our group investigated the feasibility, efficacy, and safety of xenogeneic sheep bone marrow MSC transplantation in a Wistar rat spinal cord transection model (2). We retrovirally transfected the isolated and cultured xenogeneic MSCs with the Discosoma Red Fluorescent Protein (dsRFP) gene prior to transplantation, which was performed through a mid-thoracic laminectomy and the cells relied in the form of a semi-liquid suspension with fibrin glue.

Interestingly, despite the simple model used, the lack of scaffolds and the lack of a specific injection technique, we found some evidence of engraftment of MSCs at the injury sites, confirmed by the presence of red fluorescence. The transplanted cells also showed *de novo* locally induced positivity for nestin, tubulin βIII, NG2 glia, neuron-specific enolase, vimentin and 200 kD neurofilament. In addition, the clear transdifferentiation of xenogenic MSCs into a neuroglial phenotype was able to promote a significant and durable partial functional recovery of motor functions in the rat study group (P <0.001) as assessed by the Basso-Beattie-Bresnahan locomotor scale.

We believe that the cautious enthusiasm generated by the successful xenogenic use of MSCs in SCI could act as a fulcrum to pave the way for a new line of research, theoretically able to broaden the spectrum of possible grafts for neuroregeneration.

Our group has also highlighted the enormous potential applications of stem cell therapies, especially MSCs, in the treatment of traumatic brain injury, cranial and spinal bone defects and primary malignant brain tumors, with most of the completed or ongoing trials already in phase 2 (3–5).

Immunogenicity, oncogenicity and routes of administration are still concerns, but the refinement and improvement of vector design and delivery should combine to definitively lead the transition from a purely mechanical to a biological era in the treatment of SCI.

## Author contributions

SL: Conceptualization, Validation, Writing – original draft, Writing – review & editing. AC: Supervision, Validation, Writing – review & editing. AC: Conceptualization, Validation, Writing – original draft.

## References

[1] Xia Y Zhu J Yang R Wang H Li Y Fu C . Mesenchymal stem cells in the treatment of spinal cord injury: Mechanisms, current advances and future challenges. Front Immunol. (2023) 14:1141601. doi: 10.3389/fimmu.2023.1141601 36911700 PMC9999104

[2] Luzzi S Crovace AM Lacitignola L Valentini V Francioso E Rossi G . Engraftment, neuroglial transdifferentiation and behavioral recovery after complete spinal cord transection in rats. Surg Neurol Int. (2018) 9:19. doi: 10.4103/sni.sni_369_17 29497572 PMC5806420

[3] Luzzi S Crovace AM Del Maestro M Giotta Lucifero A Elbabaa SK Cinque B . The cell-based approach in neurosurgery: ongoing trends and future perspectives. Heliyon. (2019) 5:e02818. doi: 10.1016/j.heliyon.2019.e02818 31844735 PMC6889232

[4] Giotta Lucifero A Luzzi S Brambilla I Trabatti C Mosconi M Savasta S . Innovative therapies for Malignant brain tumors: the road to a tailored cure. Acta BioMed. (2020) 91:5–17. doi: 10.23750/abm.v91i7-S.9951 PMC7975829 32608372

[5] Luzzi S Giotta Lucifero A Brambilla I Trabatti C Mosconi M Savasta S . The impact of stem cells in neuro-oncology: applications, evidence, limitations and challenges. Acta BioMed. (2020) 91:51–60. doi: 10.23750/abm.v91i7-S.9955 PMC7975826 32608375

